# Advances in Hereditary Colorectal Cancer: How Precision Medicine Is Changing the Game

**DOI:** 10.3390/cancers17213461

**Published:** 2025-10-28

**Authors:** Shenghao Lin, Chenxi Zhou, Hanlin Chen, Xinlei Zhou, Hujia Yang, Leitao Sun, Leyin Zhang, Yuxin Zhang

**Affiliations:** 1 The First Affiliated Hospital of Zhejiang Chinese Medical University (Zhejiang Provincial Hospital of Chinese Medicine), Hangzhou 310006, China; linsh@zcmu.edu.cn (S.L.); zhouchenxi@zcmu.edu.cn (C.Z.); 202212221505029@zcmu.edu.cn (H.C.); 202023210101032@zcmu.edu.cn (H.Y.); sunnylt@zcmu.edu.cn (L.S.); 2 The Third Clinical Medical School, the Rehabilitation Medical School, Zhejiang Chinese Medical University, Hangzhou 310053, China; 202212211702016@zcmu.edu.cn; 3 Academy of Chinese Medical Science, Zhejiang Chinese Medical University, Hangzhou 310053, China; 4 Key Laboratory of Neuropharmacology and Translational Medicine of Zhejiang Province, School of Pharmaceutical Sciences, Zhejiang Chinese Medical University, Hangzhou 310053, China; 5 Department of Oncology, Hangzhou TCM Hospital of Zhejiang Chinese Medical University (Hangzhou Hospital of Chinese Medicine), Hangzhou 310007, China; 6 Department of Radiation Oncology, Hangzhou Cancer Hospital, Hangzhou 310002, China

**Keywords:** HCRC, lynch syndrome, familial adenomatous polyposis, screening, treatment, assessment, artificial intelligence, precision

## Abstract

Only about 5% of colorectal cancers are hereditary, which is due to the low carrier rate of pathogenic gene mutations. The typical pattern of these cases is intergenerational aggregation within families and early onset. But public awareness of early diagnosis and intervention of HCRC is insufficient, resulting in most patients being diagnosed only after developing cancer, thereby missing the optimal window for treatment. This article reviews the latest developments in precision screening, treatment, evaluation and prevention strategies for HCRC, including innovative uses of artificial intelligence (AI) in molecular diagnostics, imaging technology advances, and potential application prospects. Regarding precision screening, tests of genomics, transcriptomics, microbiome, etc., combined with personalised risk stratification, can, respectively, effectively detect pathogenic mutations and “cancer-promoting” intestinal environments in the preclinical stage. AI combined with endoscopic and imaging tools has improved the accuracy of polyp detection and tumor profiling. Liquid biopsy and molecular marker detection provide new non-invasive monitoring solutions. In precision treatment, beyond traditional approaches like surgery and chemotherapy, immunotherapy with checkpoint inhibitors may be considered for HCRC patients with mismatch repair deficiency (dMMR). For patients harboring somatic mutations such as KRAS or BRAF V600E, targeted therapy can be guided by these specific mutations. Regarding precision assessment, AI incorporates microsatellite instability (MSI) detection and imaging diagnostic techniques, crucial for integrating genetic, environmental, and lifestyle data in follow-up. This helps assess the risk of recurrence and adjust the long-term medication regimens, as well as provide effective nutritional support and psychological counselling. In summary, the rapid development of precision medicine is driving the clinical management of HCRC into the era of tailored care, aiming to optimise patient outcomes.

## Simple Summary

Hereditary colorectal cancer usually develops at a relatively young age, but the lack of awareness of early screening results in most patients being diagnosed only after developing cancer, and the condition may affect the entire family. With the continuous integration of artificial intelligence and healthcare, artificial intelligence-driven multi-omics data parsing, deep learning image diagnosis and family genetic risk prediction models are maturing, providing technical support for the accurate identification of high-risk groups. Meanwhile, emerging strategies such as immunotherapy targeting mismatch repair defects, gene editing and microbiome intervention have opened new prospects for individualised treatment. This review aims to summarise the latest research advances in screening, treatment, prognosis and prevention of hereditary colorectal cancer to help optimise early screening strategies, improve treatment and enhance patient prognosis.

## 1. Introduction

As one of the most prevalent malignant tumors worldwide, colorectal cancer (CRC) incidence is on the rise in younger people [1]. Hereditary colorectal cancer (HCRC) is a type of CRC caused by specific heritable germline gene mutations. These mutations follow Mendelian inheritance patterns and significantly increase the lifetime risk of developing CRC in carriers. Among them, Lynch syndrome (LS) is the most common, followed by familial adenomatous polyposis (FAP), which together account for approximately 5% of CRC cases [2]. Another 20% or more of patients have a family history of CRC, but no single high-penetrance mutation has been identified, suggesting that shared genetic pathways or environmental risk factors are involved [3]. These diseases are usually more than a decade earlier than sporadic CRC and affected individuals frequently develop primary tumors or precancerous polyps [4]. According to reports, approximately 1 in 279 people in the United States carry LS-related MMR gene mutations, yet it is estimated that 98% of LS carriers are unaware of their condition [5]. UK data indicate that approximately 1 in 400 people carry LS-causing mutations, but fewer than 5% of those affected are aware of their condition [6]. This means that the vast majority of genetically susceptible individuals are often diagnosed with the genetic syndrome only after cancer has already developed. All of these factors emphasize why early screening of HCRC is so important in identifying individuals and families at significantly elevated risk, for whom tailored preventative measures can be lifesaving.

Different countries have varying strategies for identifying HCRC. The United States and the United Kingdom have implemented universal preliminary screening for all newly diagnosed CRC cases to identify patients at risk for LS. However, not all patients with abnormal screening results proceed directly to germline genetic testing. The decision to pursue definitive genetic testing involves a nuanced clinical assessment that considers factors such as patient age, tumor characteristics and family history to distinguish sporadic cases from true hereditary cases [7,8,9]. Japan and South Korea, however, still relies on family history assessment as the foundation, using the classic Amsterdam and Bethesda criteria to implement stratified screening [10,11]. China’s identification model is gradually transitioning from family-based risk screening to a strategy combining tumor molecular testing [12,13,14]. Despite differing approaches, all countries emphasise the importance of integrating family history, molecular testing, and genetic testing for the precise identification and management of HCRC.

Against this backdrop, precision medicine, a medical paradigm centered on “individual differences” is based on an individual’s genetic background, molecular phenotype, lifestyle, and clinical information to identify mutation types, achieve precise diagnosis and subtyping, and quantify risks, thereby enabling the development of personalized prevention, diagnosis, treatment, and assessment for HCRC patients [15,16]. For individuals found to carry pathogenic variants (PVs) in genes such as *MLH1*, *MSH2*, or *APC*, we can now implement targeted, early interventions to reduce cancer incidence and improve long-term survival [10,17,18]. The treatment paradigm for HCRC is progressively shifting towards individualized strategies, with microsatellite instability (MSI) and dMMR assays based on molecular tumor subtype classification to aid in the diagnosis of LS and to identify patients eligible for immune checkpoint inhibitor therapy, somatic biomarkers, such as *KRAS* and *BRAF V600E*, are primarily used to distinguish between sporadic and MSI-H HCRC and can guide targeted therapy [19,20]. In addition, personalized surgical and chemopreventive interventions, such as prophylactic colectomy and aspirin prophylaxis, can be implemented based on the genetic risk characteristics of LS and FAP [21,22]. Overall, genomic diagnosis and precision interventions are markedly improving the prognosis of HCRC.

Thanks to the growing availability of data resources in the medical field and technological breakthroughs in artificial intelligence (AI) for complex data analysis and automated decision-making, AI is emerging as an adjunct in HCRC care [23]. AI-assisted colonoscopy systems can achieve higher adenoma detection rates and significantly reduce missed diagnosis rates [24,25]. In the fields of pathology and imaging, deep learning tools are being developed for automate tumor classification and help predict patient outcomes [26,27]. Separately, machine learning models that combine a person’s clinical history, lifestyle, and genetic profile are promising to identify high-risk individuals more accurately, providing low-cost, rapid tools for screening high-risk populations [28,29,30,31]. These approaches promise to improve the accuracy of diagnosis and treatment decisions for HCRC.

In conclusion, benefiting from the rise of AI, precision medicine has great potential to improve the clinical management of HCRC, and we are expected to reduce the burden of this disease further. The aim of this review is to provide an overview of how current advances in precision medicine are improving all aspects of HCRC, from diagnosis to treatment to prognosis and prevention, and to discuss future perspectives.

## 2. Precision Screening Strategies

### 2.1. Genomics

The diagnosis of HCRC has shifted from relying on clinical features such as familial clustering and early-onset tumors to genome-driven universal screening [32]. Whole-tumor testing and whole-patient genetic testing are being gradually implemented. Next-generation sequencing (NGS) technology enables multi-gene panel testing (MGPT), which not only detects known susceptibility genes but also identifies rare pathogenic genes and low-to-moderate-risk mutations in genes [33,34]. For high-risk individuals with a family history or polyposis syndromes, early and frequent colonoscopy follow-ups are often clinically recommended, alongside NGS multi-gene testing [35]. Circulating tumor DNA (ctDNA), a liquid biopsy biomarker, can be detected before clinical symptoms appear. Its sensitivity often surpasses traditional methods, such as imaging, and it enjoys high patient acceptance, demonstrating significant potential in early HCRC detection and assessment [36,37]. Breakthroughs in genomics are progressively advancing HCRC screening and diagnosis toward precision and personalisation, offering hope for early disease intervention and treatment.

#### 2.1.1. Multigene Panel Testing

The methods for detecting HCRC have undergone rapid evolution in recent years. Next-generation sequencing-based MGPT has recently broadened HCRC risk assessment beyond the classic LS and FAP genes [38,39]. Studies indicate that approximately 10–15% of CRC patients carry germline PVs, many of which are heritable mutations that may be missed under phenotype-driven screening criteria. Over half of these germline variants are located outside traditional single-syndrome genes [40]. MGPT can detect variants in genes with moderate penetrance or atypical susceptibility, such as *ATM*, *CHEK2*, and *BRCA2*, significantly improving variant detection rates to approximately 36% and driving screening strategies from phenotype-driven to genotype-driven [41,42]. Interpreting results from MGPT requires a deep understanding of each gene’s role in causing cancer. Table 1 summarizes several key genes commonly found in HCRC MGPT and their functions.

The degree of dominance varies significantly among different HCRC genes. FAP driven by the *APC* gene exhibits near-complete dominance, while the *MUTYH* gene carries an extremely high risk of approximately 70–90% [63]. In contrast, MMR genes associated with LS exhibit gene-specific risks. Carriers of *MLH1*/*MSH2* mutations exhibit a CRC penetrance rate of over 40%, which is higher than the rates of 20% or less for *MSH6* or *PMS2* [72,73]. Extensive prospective data similarly confirm that *MLH1*/*MSH2* mutations confer a high risk of CRC, while *MSH6* mutations only slightly increase the risk, and *PMS2* shows no clear CRC penetrance [74]. Genes with moderate penetrance, such as *CHEK2* and *ATM*, carry only a low risk of CRC [47]. (Currently, there are no established risk grading standards for HCRC genes. Based on an extensive literature review, we classify columns with CRC incidence rates above 50% as high risk, those with an incidence below 10% as low risk, and the remainder as medium risk). In addition, some HCRC patients carry two or more PVs, with an incidence rate of approximately 1% to 2% [75]. These mutations often involve combinations of classic high-penetrance genes (such as *MLH1* and *MSH2*) and moderate-penetrance genes (such as *CHEK2* and *ATM*) [76,77,78]. Research indicates that the concurrent occurrence of multiple gene mutations can form synergistic carcinogenic effects at the molecular level. Specifically, *MLH1* mutations lead to the loss of MMR function, while *ATM*, a key kinase in the DNA double-strand break damage response, undergoes mutations that weaken cell cycle checkpoints and homologous recombination repair pathways. The loss of functional complementarity between the MMR pathway and the HRR pathway significantly increases genomic instability [79,80,81]. *MSH2* mutations cause defects in DNA MMR, while *CHEK2* mutations weaken cellular control of DNA double-strand break checkpoints and repair signaling, resulting in the loss of functional complementarity between DNA repair pathways [82,83,84]. Additionally, clinical observations indicate that carriers of such mutations often exhibit earlier onset age, a broader tumor spectrum, and a tendency toward multiple primary cancers, suggesting that polygenic mutations may be positively correlated with more complex clinical manifestations [84,85]. Therefore, the identification of multiple gene mutations is of some significance for HCRC risk assessment and the formulation of personalized intervention strategies.

Based on these advances, the 2024 NCCN guidelines now recommend performing MGPT for all CRC patients to identify potential HCRC risk [35,53]. Still, how MGPT should be applied clinically and its practical value needs further clarification [86]. Studies have shown that the detection rate of hereditary PVs is significantly higher in patients with early-onset HCRC compared to those with late-onset HCRC. Approximately 21.8% of CRC patients under 50 years of age carry pathogenic or likely pathogenic germline variants, while the positive rate in the 50-year-old and older group is approximately 12.2% [87,88]. Additionally, the detection rate of PVs in rectal cancer patients is slightly higher than in HCRC patients (15.3% vs. 10.5%), suggesting that the tumor site may also play a role [89,90,91]. Moreover, family history remains an important indicator of genetic risk. Patients with a family history of CRC have a pathogenic variant detection rate as high as 31%, significantly higher than those without a family history [92,93]. Also, the MGPT results vary across different racial and ethnic groups. Studies indicate that among patients with early-onset CRC under the age of 50, approximately 12.4% of White patients carry germline PVs, 10.3% of Black patients, 9.5% of Asian patients, Hispanics 14.0%, and Ashkenazi Jews 12.7%, though the overall favourable rates may be similar [94,95,96]. In summary, the application of MGPT in HCRC must consider factors such as patient age, family history, and race. Comprehensive MGPT is especially recommended for younger HCRC patients with a family history.

#### 2.1.2. ctDNA Detection

Liquid biopsy, as a potential non-invasive detection method, has emerged as a promising approach for early CRC detection in LS patients [97]. Due to the near-universal presence of MSI driven by dMMR in LS-associated CRC, MSI serves as an ideal target for liquid biopsy-based detection of HCRC. In contrast, tumors arising from other genetic syndromes, such as FAP or *MUTYH*-associated polyposis, are typically microsatellite stable (MSS). This typically involves analyzing changes in the length of microsatellite repeats in ctDNA or calculating MSI scores via NGS [98,99,100]. In LS carriers undergoing colonoscopy surveillance, blood-based ctDNA testing can help identify interval cancers or advanced adenomas that colonoscopy may miss, thereby improving compliance by reducing reliance on invasive procedures [101,102]. Recent studies have shown that LS-associated tumors release characteristic ctDNA alterations, including somatic mutations in MMR genes and high-burden insertion-deletion mutations reflecting dMMR mutations [103]. This provides a clear way to distinguish them from sporadic CRC. In sporadic cases, even when the tumor is microsatellite-high (MSI-H), the underlying cause is rarely a germline mutation. The underlying cause is typically somatic hypermethylation of the *MLH1* promoter. This explains why a high proportion (69–78%) of these sporadic MSI-H tumors carry the *BRAF V600E* mutation, which sharply contrasts LS tumors, where the mutation is rarely found [104,105,106]. This implies that detection of the *BRAF V600E* mutation in ctDNA strongly suggests sporadic MSI tumors, while its absence points to LS.

Currently, the use of plasma ctDNA testing for MSI detection to monitor LS-related risks remains limited but shows significant potential. Preliminary data indicate that MSI-related mutation patterns can be detected in the blood of LS patients with advanced cancer. Current research is evaluating its sensitivity to early-stage tumors, with one key limitation being that early-stage LS lesions may release extremely low levels of ctDNA [107]. Recent technological advances have enabled highly sensitive detection of MSI in plasma. Silveira et al. demonstrated that digital polymerase chain reaction (PCR) could accurately determine the MSI status of ctDNA, consistent with tumor tissue results [108]. More recent studies have combined NGS technology with the bioinformatics tool MSIsensor to detect MSI in plasma by identifying the whole-genome microsatellite mutation burden [109,110]. Despite some challenges, ctDNA-based monitoring holds significant potential in LS, complementing colonoscopy to detect extra-colonic LS tumors such as endometrial cancer and ovarian cancer and serving as an “early warning signal” for malignant tumors between colonoscopies.

### 2.2. Other Multi-Omics Technologies

The integrated application of multi-omics technologies is driving innovation in the screening of HCRC by comprehensively analyzing genetic susceptibility, molecular characteristics, and microenvironmental changes [111]. Transcriptomics further reveals the characteristic expression profiles of DNA dMMR intestinal stem cells: for example, in LS models, dMMR stem cells exhibit suppressed cell replication and Wnt signalling pathways, as well as activated epithelial signalling and immune response pathways [112,113]. Their unique gene expression signatures can reproduce the early steps of tumor development and may serve as novel early biomarkers [111]. Meanwhile, proteomics and metabolomics play a crucial role in the development of non-invasive biomarkers: analyses of plasma, faeces, and urine have identified a series of early screening candidate markers, including traditional tumor antigens such as CEA and CEACAM family proteins, as well as abnormal changes in metabolic product profiles (e.g., elevated bile acids and dysregulated short-chain fatty acid ratios), providing clues for early detection [114,115,116,117]. Gut microbiome research has revealed unique differences in the gut microbiota community structure between LS carriers and healthy individuals [118]. The study found that certain symbiotic bacteria, such as *Faecalibacterium prausnitzii*, *Parabacteroides distasonis*, and *Bacteroides fragilis*, are significantly enriched in the guts of LS patients and exhibit dysbiosis characteristics related to their genetic background [119,120,121].

Combining multi-omics technologies provides a deeper comprehension of HCRC pathogenesis, establishing a new scientific basis for precision screening. Through multi-omics network analysis (e.g., MUFFIN) integrating microbial and host transcriptomic data, correlations between specific microbial abundance and host gene expression were observed—the enrichment of the pathogenic bacterium *Campylobacter jejuni* was found to be associated with the activation of colonic mucosal inflammatory responses and DNA damage response pathways—thereby revealing the pro-inflammatory carcinogenic role of the microbiota in tumor development [122,123]. Emerging spatial transcriptomics and single-cell omics are powerful new tools for dissecting the microenvironment and heterogeneity of HCRC [124]. These techniques allow researchers to construct multi-omic maps of LS-associated lesions, which in turn lets them identify distinct cell populations, their spatial arrangements, and their interactions during early tumor development [125,126]. For instance, single-cell analysis has found that cancer stem cell-like populations with high CEACAM5 expression are already present in the normal-appearing mucosa of LS patients. Furthermore, LS tumors exhibit unique patterns of immune cell infiltration and mutation burden. These findings give us a much deeper understanding of the microenvironmental ecology and evolutionary mechanisms of early carcinogenesis [127]. In summary, multi-omics integration has significantly advanced the early screening, biomarker discovery, and mechanistic understanding of HCRC, providing a new scientific foundation for precise monitoring and intervention in high-risk populations.

### 2.3. Artificial Intelligence

**Figure 1:**
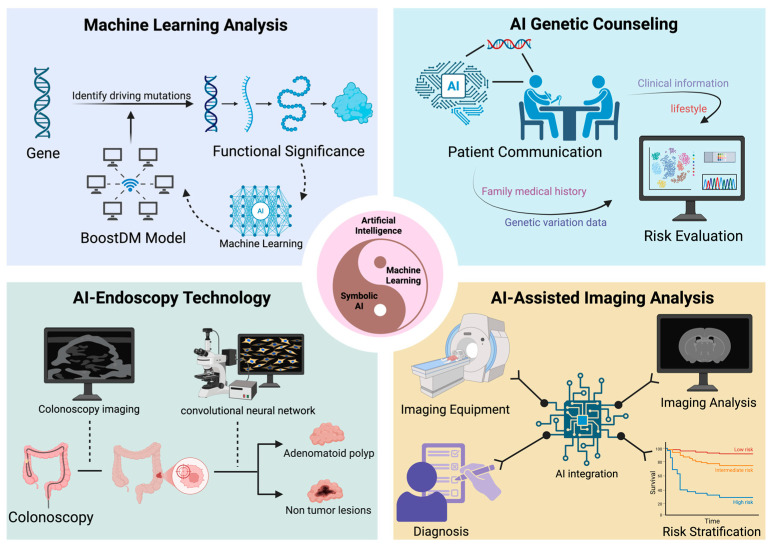
The application of AI in HCRC screening.

AI has quickly become a transformative tool in HCRC screening. Its applications range from genetic risk assessment to endoscopic image analysis [128]. In addition, machine learning and deep learning models are working to solve the long-standing challenge of interpreting variants in NGS data [129]. Specific applications are listed in Figure 1.

#### 2.3.1. AI in Genetic Counseling and Cancer Risk Prediction

The rise of AI is dedicated to addressing the long-standing challenges of interpreting variations from NGS data. The BoostDM model uses machine learning to perform in silico saturation mutation analysis of cancer genes, improving the sensitivity of identifying PVs in unscreened HCRC patients and predicting the potential functional significance of certain intronic variants [130,131]. It provides interpretable carcinogenicity scores for rare variants [132]. Such AI tools have been integrated into interpretation pipelines to help uncover hidden carcinogenic variants in genomic data. As the number of patients undergoing multi-gene testing increases, AI-based chatbots have been used to provide preoperative education and risk assessment for hereditary cancers [133,134]. Studies have shown that AI chat tools are comparable to traditional counselling in terms of patient preoperative consultation completion rates and genetic testing acceptance rates [135]. This demonstrates their potential to expand access to genetic services and reduce the burden on genetic counsellors. In risk prediction, polygenic risk scores combined with clinical risk models such as QCancer-10 enhance the predictive performance for HCRC incidence. The C-statistic—used to evaluate the accuracy of binary prediction models—increased from 0.69 to 0.73 (with values ranging from 0.5, indicating no predictive ability, to 1, indicating perfect prediction). Prediction results indicate that the incidence rate in the high-risk group is 2–3 times that of the moderate-risk group [136]. An extensive cohort study demonstrated that machine learning models (e.g., LightGBM), integrating factors such as age, family history, and lifestyle, achieved an Area Under the Receiver Operating Characteristic Curve value of 0.73. (Typically, values above 0.7 are considered indicative of good predictive performance, enabling more precise risk stratification and treatment planning.) This translates to successfully distinguishing high-risk individuals from average-risk individuals 73 times out of 100, showcasing the potential for personalized screening [137]. Finally, an analysis of ChatGPT-4 and similar large language models in the medical field indicates that these models can explain genetic concepts in layperson’s terms and engage in personalized communication in genetic counselling [138,139]. A systematic review highlights that ChatGPT demonstrates remarkable efficiency in generating educational materials, answering patient questions, and assisting with documentation [140]. As it stands, AI models fall short of replicating the empathy or navigating the ethical subtleties of patient counselling, and crucially, they carry the risk of introducing or amplifying data bias [141]. Although GPT-4-based chatbots have demonstrated feasibility in pilot projects, the current consensus is that they should be used as an adjunct to human counsellors rather than a replacement [142].

#### 2.3.2. Advanced Endoscopic, Imaging Technology and AI-Assisted Diagnosis

Effective screening for HCRC hinges on advanced endoscopic techniques and diagnostic systems [143]. Specifically, combining tools like high-resolution endoscopy, narrow-band imaging (NBI), and chromoendoscopy can greatly enhance adenoma detection rates and, together with AI-aided detection and diagnosis systems, allow for real-time assessment of lesion malignancy [144]. For example, employing chromoendoscopy for HCRC screening can elevate the adenoma detection rate to 30%, representing a significant improvement over the 21% detection rate achieved with standard high-definition white-light endoscopy. This advancement contributes to further enhancing the early diagnosis rate of HCRC [145]. AI-driven image analysis can also predict the depth of tumor infiltration and determine if a lesion has breached the submucosal layer, providing critical evidence for treatment decisions such as endoscopic resection or surgical intervention [146].

In patients with HCRC, AI-assisted CT colonography significantly enhances the detection rate and characterization of colorectal lesions while enabling patient stratification based on malignant risk [147,148]. A deep learning “Faster R-CNN” network trained on a multicenter CT colonography dataset demonstrated 82% sensitivity for lesions ≥6 mm compared to manually annotated lesion regions in internal validation, suggesting the model’s potential for optimizing HCRC screening workflows [149]. In radiomics approaches, automated extraction of CT colonography features enables precise differentiation between hyperplastic and adenomatous polyps in HCRC patients, guiding resection decisions [150]. However, it is essential to note that this technology cannot replace sound clinical judgment. In the context of high-risk hereditary conditions, nearly all detected polyps are resected due to elevated cancer risk. Emerging evidence suggests that a “diagnosis-and-wait” strategy may be feasible for rectosigmoid polyps ≤5 mm. Experienced endoscopists employing high-confidence optical diagnosis achieve a 96% negative predictive value for benign histology, potentially avoiding approximately 59% of polypectomies [151]. This comes at the cost of missing a small number of microadenomas. Therefore, we explicitly state that deferring immediate resection for some micro-polyps is only appropriate within research or expert monitoring programs, and this approach remains controversial rather than a standard practice. Similar deep learning methods in abdominal MRI are still in the preliminary research phase [152]. The comprehensive application of AI systems helps standardize screening processes, reduce operator variability, and enhance examination consistency, thereby improving the overall quality of screening.

## 3. Precision Treatment Strategies

### 3.1. Surgical and Endoscopic Interventions

Deciding on the right surgical approach for patients with HCRC requires careful consideration of their specific genetic profile and cancer risk [153]. For individuals with LS who haven’t yet developed cancer, we generally don’t recommend a preventative total colectomy. Consistent colonoscopy screenings and removing polyps as they appear can effectively keep cancer at bay for a significant time [154]. Once cancer does develop in an LS patient, the decision on how extensive the surgery should be depends on their specific genetic mutation and the likelihood of a recurrence. For those carrying mutations in the *MLH1* or *MSH2* genes, a more extensive colectomy is often considered during the first surgery to lower the chances of a new cancer developing later on [155]. On the other hand, for patients with *MSH6* or *PMS2* mutations, the risk of recurrence is lower, so a more limited resection that just removes the tumor is usually enough [156]. For women with LS, considering the increased risk of secondary endometrial and ovarian cancers, concurrent risk-reducing surgery is recommended after childbearing is complete [157]. The situation is different for patients with FAP, prophylactic surgery is typically required during adolescence due to the high burden of colonic polyps, which almost invariably progress to cancer [158,159]. Patients with attenuated FAP, often treated with total abdominal colectomy and connect the ileum to the rectum. For those with the more severe, classic form of FAP, a total proctocolectomy with the creation of an ileal pouch is the preferred approach [160].

Intensive endoscopic surveillance and minimally invasive interventions are vital for precision prevention in high-risk HCRC populations [161,162]. With the continuous development of endoscopic techniques, small polyps and early-stage intramucosal cancers can be effectively resected using endoscopic mucosal resection or endoscopic submucosal dissection, thereby avoiding major surgery [163]. For early-stage tumors confined to the submucosa, such approaches have the advantage of being less invasive and preserving bowel function [164]. Endoscopic polypectomy achieves a 90.5% complete resection rate in FAP-associated small bowel tumors [147].Robotic-assisted surgery is emerging as superior to laparoscopic surgery for HCRC. A single-center study comparing robotic-assisted versus laparoscopic left hemicolectomy showed that robotic-assisted surgery significantly reduced hospitalization time (6 days vs. 10 days, *p* = 0.025) and postoperative complications (8.6% vs. 0%, *p* = 0.126) [165]. Ultimately, the modern strategy for managing HCRC is centered on being proactive. This means focusing on early detection and intervention, whether that involves preventive surgery to lower cancer risk from the start, or diligent surveillance and minimally invasive removal of any suspicious growths before they can become a bigger problem.

### 3.2. Medication

Pharmacotherapy is an important part of the precision treatment of HCRC, and its strategy has evolved from traditional broad-spectrum chemotherapy and chemoprophylaxis to specific drugs for specific molecular targets and immunotherapies with better efficacy for specific immune phenotypes, forming specific treatments for different genetic syndromes, and new drugs or combinations are constantly emerging from clinical trials [166,167,168].

#### 3.2.1. Chemoprevention

In the field of chemotherapy, aspirin is widely recommended as a chemopreventive agent for individuals with LS. Metabolomic analyses have revealed that aspirin prophylaxis exerts beneficial effects on platelet activation, vascular function, and inflammatory status through modulation of systemic prostaglandin biosynthesis [169]. Evidence from the CAPP2 trial and its long-term follow-up indicates that daily administration of 600 mg aspirin for over two years can reduce the long-term risk of CRC by more than 60% [167]. In FAP, the primary aim of chemoprevention is to induce polyp regression and delay surgical intervention. NSAIDs such as sulindac and celecoxib have shown efficacy, though their benefits are transient [170,171]. Tumor-related metabolic network studies have shown that COX and ODC activities are frequently elevated in FAP. By reducing polyamine levels, sulindac and celecoxib can decrease both the size and number of adenomas via COX-dependent or COX-independent mechanisms, although their overall preventive efficacy remains limited [170,171,172]. Extensive transcriptomic studies have also demonstrated that estradiol can influence the proliferative profile of LS-associated endometrial carcinoma (LS-EC) cells by regulating PgR expression through endoplasmic reticulum pathways [173]. For women with LS, estrogen-only hormone replacement therapy initiated before the age of 51 is now recognized as a consensus strategy to reduce the risk of endometrial cancer [174]. As for adjuvant chemotherapy, several studies have demonstrated that patients with stage II dMMR/MSI-H HCRC not only fail to benefit from 5-fluorouracil-based regimens but may also be harmed by such treatments. It is therefore essential to avoid unnecessary exposure to the toxicity of ineffective therapies in this subgroup [175].

Bioinformatics provides critical support for the design of novel drugs and drug combinations. For example, pan-cancer analysis using the TCGA database revealed that autophagy-related protein 5 plays a key role in tumor metabolism and immunity, is closely associated with *MSH2* expression and the apoptosis/autophagy pathway, and has been identified as a potential new immunotherapy target for LS patients [176]. TCGA’s whole-exome sequencing data have enabled the design of cancer vaccines targeting new antigens generated by recurrent frameshift mutations in MSI-H tumors, such as shared frameshift peptide vaccines and dendritic cell vaccines, which can induce strong T-cell responses [177]. Additionally, spatial genomics studies have further guided the development of such vaccines by incorporating multiple frameshift peptide epitopes from different tumor clonal origins into the design [178]. At the transcriptomic level, a cohort study identified MSH4 as a potential new therapeutic target in LS-associated tissues; TCGA data analysis also showed that high MSH4 expression is associated with increased tumor mutational burden and poor overall survival (OS) outcomes [179]. Meanwhile, the discovery of new mutation mechanisms has revealed the limitations of traditional therapies: frameshift mutations observed in LS patients lead to truncated *MSH6* protein and splicing defects, rendering 5-fluorouracil-based adjuvant chemotherapy ineffective in such patients [180]. This also suggests that the expanding spectrum of biomarkers is increasingly complicating decision-making for molecularly stratified therapies [181]. In summary, the ability of bioinformatics to integrate large-scale molecular data with clinical outcomes holds promise for further optimizing precision drug development.

#### 3.2.2. Molecular Targeted Therapy

In patients with HCRC, if the tumor carries a specific driver gene mutation or susceptible molecular target, corresponding targeted therapies may be considered. Studies have shown that a dual-targeted combination of the oral BRAF inhibitor enkephalinib and the anti-EGFR monoclonal antibody cetuximab prolonged median OS from 5.9 to 9.3 months in them, establishing this combination as the standard of care [182]. Although mutations in the *RAS* gene (*KRAS*/*NRAS*) were once considered a contraindication to anti-EGFR therapy, targeted inhibitors such as sotolacib and adaglacib for patients with *KRAS G12C* mutations have shown potential, with objective remission rates (ORR) of approximately 19%, rising to 46% when combined with cetuximab, and median progression-free survival (PFS) approaching 7 months [183]. For *HER2*-amplified, *RAS*/*BRAF* wild-type metastatic HCRC, the combination of the *HER2* tyrosine kinase inhibitor trastuzumab with tucatinib demonstrated a 38.1% response rate in patients resistant to standard therapy, with some patients achieving complete remission [184,185]. Though rare in HCRC, *NTRK* gene fusions are actionable targets for TRK inhibitors, which have shown durable efficacy and favorable safety profiles [186]. While *HER2* amplification and *BRAF* mutations are infrequent in LS-associated tumors, they may still occur in other hereditary syndromes, such as *MUTYH*-associated polyposis [187,188]. Table 2 summarizes the current molecular targeted therapies.

Overall, molecular profiling for targets such as RAS, BRAF, HER2, and NTRK is essential for guiding targeted therapy. Once drug-sensitive mutations are identified, incorporating targeted therapy into treatment regimens is critical for individualized care.

#### 3.2.3. Immunotherapy

The emergence of immune checkpoint inhibitors has significantly altered the therapeutic landscape of HCRC. LS-associated tumors, due to their high mutational load and MSI, show a strong response to PD-1/PD-L1 inhibitors, thanks to their ability to generate large amounts of neoantigens [189,190]. Clinical trials found that in patients with MSI-H/dMMR HCRC who are refractory to therapy, first-line treatment with pembrolizumab prolonged median progression-free survival from 8.2 to 16.5 months, reduced the risk of disease progression or death by 41%, and resulted in significantly lower toxicity compared to standard chemotherapy [191]. Cercek et al. reported that the PD-1 inhibitor neoadjuvant immunotherapy with dostarlimab resulted in complete tumor remission in all patients with dMMR, locally advanced rectal cancer, offering the possibility of avoiding surgery in highly immune-responsive individuals [168]. A combined strategy of PD-1 and CTLA-4 blockade showed superior efficacy to monotherapy. Study discovered that combining ipilimumab and nivolumab increased the objective response rate of MSI-H/dMMR HCRC from 28% to 46% [192].

Most LS exhibit dMMR and high immunogenicity, which explains their significant response to PD-1 checkpoint inhibitors [193]. However, a minority of LS cases remain microsatellite stable or exhibit low immune activation, indicating a poor response to immunotherapy [194]. In such cases, immune checkpoint inhibitors alone are insufficient for preventing tumor-specific neoantigens in HCRC. Combination immunotherapy strategies are being explored, and neoantigen vaccines have been proposed as prophylactic interventions for LS. A clinical trial that utilized transposons embedded within a neoantigen vaccine revealed that 91% of patients exhibited an augmented immune response subsequent to treatment with an immune checkpoint inhibitor [195,196]. Based on aspirin’s immunomodulatory effects, the University of Texas MD Anderson Cancer Center conducted a matched pre- and post-treatment chemoprevention trial using naproxen. Analysis of 36 samples revealed significant changes in immune cell populations and demonstrated anti-inflammatory effects comparable to those of aspirin, suggesting potential synergistic effects with immune checkpoint blockade [197,198]. An 8-week dose of the histone methyltransferase EZH2 inhibitor GSK503 significantly increased tumor-infiltrating CD4+ and CD8+ T cell populations [199]. In addition, gemcitabine in combination with a PD-L1 inhibitor showed potential to enhance tumor immunogenicity in low-immunogenic HCRC. In mouse models, 33% of *MLH1* knockout mice remained tumor-free at least 65 weeks after ultra-low-dose cyclophosphamide chemotherapy [200,201]. Additionally, combination therapies, such as regorafenib plus nivolumab, achieved an approximately 33% ORR in MSS HCRC patients by reducing FoxP3+ regulatory T cells in the tumor microenvironment [202]. Future precision oncology studies should aim to extend the benefits of immunotherapy from the MSI-H population to a broader group of patients with HCRC.

### 3.3. AI-Integrated Personalized Treatment

Precision treatment depends not only on advanced medications and interventions but also on an efficient collaborative system that integrates data from multiple sources, coordinates interdisciplinary experts, and supports complex decision-making. The treatment of HCRC involves a wide range of specialties, including prevention, endoscopy, surgery, chemotherapy, immunology, and genetic counseling. In this context, the multidisciplinary consultation (MDT) model becomes a critical link that helps improve patient adherence and satisfaction with treatment [103,104]. The MDT model is also particularly suitable for managing complex cases, such as the presence of multiple primary tumors in the same period of time, the need for combined pelvic organ resection or organ transplantation evaluation, etc., to reduce the complication rate [105,106]. In addition, the intervention of AI further enhances precision, with its strength in extracting patterns that are difficult for humans to detect from large amounts of complex data, enabling more accurate prediction of treatment response as well as the development of personalized treatment strategies, changing the way HCRC is managed [107]. For example, Chen et al. used PET-CT images to train a model that can predict the degree of inflammation in the tumor microenvironment and the patient’s response to PD-1 inhibitors prior to immunotherapy, allowing early differentiation of potential beneficiaries and optimization of the timing of intervention [108]. In addition to single modality, multimodal AI fuses data from different sources for analysis to provide more comprehensive predictions. In the latest study, by feeding pathology images, gene sequencing and clinical data into a deep learning model, it can be used to predict the efficacy of immune checkpoint inhibitors in patients with gastrointestinal tumors [109]. Frameshift-derived Neoantigen genes with optimal coverage in LS carriers can also be predicted, providing a potential opportunity for dMMR HCRC to develop frameshift peptide neoantigen vaccination [110]. In conclusion, AI is gradually being integrated into the whole management of HCRC precision therapy, showing potential from assisted decision-making to efficacy prediction, which is expected to enhance individualized management.

## 4. Precision Prevention and Assessment Strategies

### 4.1. Pre-Tumor: Lifestyle and HCRC Prevention

The core of effective HCRC prevention lies in integrating genetic risk stratification with personalized interventions. Current recommendations involve implementing precision strategies after identifying mutation carriers, including enhanced colonoscopy protocols for high-risk genotypes, chemoprevention for syndromes with proven benefits, and lifestyle guidance to optimize modifiable factors [169,203]. For LS patients, most guidelines now suggest screening around age 25 for *MLH1*/*MSH2* carriers and around age 35 for *MSH6*/*PMS2* carriers [55,162]. FAP carriers require highly intensive monitoring. Any patient with 20 or more adenomas should undergo *APC* gene testing; mutation-positive individuals should initiate colonoscopy between the ages of 10 and 15 and continue it annually thereafter [35,204]. Juvenile polyposis syndrome patients should begin colonoscopic surveillance during childhood or adolescence, with specific timing determined by mutation type and family history. In clinical practice, colonoscopies are typically performed every 1–3 years starting at age 10 [205]. Prevention for Peutz–Jeghers Syndrome focuses on hamartomatous polyps throughout the gastrointestinal tract, usually involving colonoscopies every 2–5 years starting in adolescence, along with gastroscopies every 1–2 years [206]. Lifestyle factors are equally important. High-risk individuals should adhere to standard CRC prevention recommendations: maintaining a healthy weight, consuming a high-fibre diet, engaging in regular exercise, limiting alcohol consumption, and quitting smoking [207,208]. Additionally, patients should undergo relevant cancer screenings per high-risk guidelines, such as pancreatic cancer MRI, breast MRI, and cervical cytology screening. However, these screenings fall outside the scope of CRC prevention.

### 4.2. Post-Tumor: AI-Powered HCRC Prognosis Assessment

The prognosis of HCRC patients is highly dependent on histopathological analysis and molecular marker detection [209]. The classical pathological method involves the manual interpretation of tumor heterogeneity, invasion depth, and lymph node metastasis under a microscope [210]. However, this method is slightly subjective and prone to differences in interpretation among pathologists [211,212]. In addition, with the increasing amount and complexity of pathological data, the accuracy of traditional methods in efficacy assessment and prognosis prediction is hitting a bottleneck [213].

#### 4.2.1. AI and Digital Pathology

Digital pathology employs whole slide imaging (WSI) technology to transform tissue sections into high-resolution digital images, thereby establishing a fundamental framework for standardized storage, remote sharing, and in-depth analysis of pathological data [214]. However, it must be noted that the advent of digitization in itself does not guarantee the supersession of the limitations of traditional analysis with regard to efficiency and consistency [215]. In light of these developments, AI is being integrated with digital pathology and radiology to enhance the prognostic assessment of HCRC. A salient factor in this regard is MSI status, a critical biomarker in CRC with substantial ramifications for diagnosis, treatment, and prognosis. Tumors exhibiting MSI-H, such as LS, have been observed to frequently co-occur with substantial tumor-infiltrating lymphocytes and a robust immune microenvironment. These characteristics are often associated with a more favorable prognosis than MSI-negative tumors [216]. Therefore, precise detection of MSI status is imperative. Conventional methods depend on immunohistochemistry (IHC) or PCR. Concurrently, AI presents a novel methodology, the utilization of routine patient data for the detection of MSI in vitro, thereby achieving MSI status prediction and pathological feature identification [217]. Saillard et al. demonstrated that neural networks have the capacity to accurately detect MSI in H&E-stained tumor images [218]. Follow-up studies have confirmed the high accuracy of AI in predicting MSI status, and its constructed classifiers can achieve an area under the curve (AUC) value of more than 0.9, which is promising as a powerful complement to traditional PCR or IHC assays [219,220]. In addition, bioinformatics tools, such as MSINET, utilise architectures like convolutional neural networks, which are capable of learning patterns of MSI and determining their status directly from NGS data [221,222]. As these tools continue to gain clinical validation and become more mature, they are expected to fit naturally into existing pathology workflows and become a powerful aid in assessing HCRC prognosis.

In addition to predicting the prognosis of HCRC through MSI status, tumor budding also has high clinical value. Tumor budding is an important pathological marker of the invasiveness of CRC, manifested as the presence of isolated or small clusters of tumor cells (<5 cells) at the tumor edge [223]. AI models can automatically complete high-throughput identification and counting of budding through the recognition and target detection of boundary regions in WSI images combined with morphological analysis. This can improve efficiency and reduce the subjectivity of manual assessments [224]. In a screened cohort of patients with stage II CRC, high-grade TB independently predicted recurrence [225]. In locally advanced rectal cancer treated with neoadjuvant therapy, TB still showed significant association with worse OS and disease-free survival [226]. In pT1 CRCs, TB is strongly predictive of lymph node metastasis, potentially guiding decisions on additional surgery [227]. Furthermore, combining TB with Immunocore has been shown to improve prognostic stratification in a cohort of 654 I–III stage CRCs [228]. In addition, combining immune scoring with tumor bud formation is expected to improve the prediction of disease-free survival in patients, but it is still in the experimental stage [226]. Based on these findings, we believe it holds potential as a useful adjunct prognostic marker in the HCRC cohort and for MSI status, though further dedicated studies are still required.

#### 4.2.2. AI and Image Assessment

Beyond pathologic imaging, the combination of AI and radiomics is disrupting the traditional approach to noninvasive prognostic assessment. Deep mining of CT, MRI, and PET scan data allows for the extraction of quantitative features that are closely related to tumor biology and clinical outcomes [229]. For example, AI algorithms analyzing enhanced CT images of HCRC can predict MSI status with high specificity, suggesting their potential as a screening tool that offering a potential non-invasive screening tool for LS-associated tumors [230,231]. Some studies have combined CT image features with Lasso regression models to successfully predict OS and recurrence-free survival in stage HCRC patients, achieving AUC values of 0.768 and 0.744 [232]. AI technology can also quantify skeletal muscle index and skeletal muscle area based on CT imaging and determine thresholds based on gender differences to improve the diagnostic accuracy of dystrophy and muscle atrophy, setting the stage for the implementation of personalized nutritional interventions, which are critical for improving patient prognosis [233]. Although still in its early stages, AI-enhanced imaging technology holds promise when combined with genomic and pathological data to build more comprehensive prognostic assessment models.

#### 4.2.3. Other Potential Applications

Beyond using digital pathology and imaging to assess prognosis, AI is beginning to demonstrate broad application prospects in other areas of supporting HCRC patient prognosis. Nutritional management can help ease the malnutrition and muscle loss that come with cancer and its treatments [234]. Psychological intervention, on the other hand, can reduce patients’ anxiety and depression, lifting their emotional state [235]. What’s key is that AI’s role in these areas has made it possible to actually roll out personalized nutrition and mental health plans. For example, AI-powered virtual nutritionists can create dietary plans tailored to a patient’s clinical stage and metabolic profile, helping to promptly counteract the effects of malnutrition and muscle wasting [236]. For HCRC patients face physical and psychological challenges during the diagnostic and postoperative phases, especially those who need to adapt to a new stoma. Patients are prone to anxiety and depression due to problems such as fluid leakage and odor [237,238]. Therefore, providing psychological intervention can help alleviate emotional disorders, restore physical function, reduce fatigue, and improve quality of life [239]. A recent multicenter randomized controlled trial showed that a digital psychological counseling system built with ChatGPT could significantly reduce patients’ anxiety and depression levels. HADS scores were better than those of the conventional health education group [240]. Overall, these AI-driven prognostic support tools show promise in improving the quality of care for HCRC patients.

## 5. Conclusions and Prospect

Despite progress in managing HCRC through precision medicine, key challenges persist that hinder its widespread adoption. First, HCRC exhibits intratumoral heterogeneity, with cancer cells within the same tumor harboring distinct genetic mutations, gene expression differences, and cellular phenotypic variations [241,242,243]. Consequently, molecular characterization of tumor tissue obtained from a single lesion may not fully represent the systemic disease [243]. In autosomal dominant HCRC, PVs may occur in multiple DNA MMR genes (e.g., *MLH1*, *MSH2*, *MSH6*, *PMS2*) [6,35,244]. In contrast, FAP is primarily caused by germline mutations in the *APC* gene, but the location and type of mutations significantly influence the phenotype [245,246]. This potential genetic diversity leads to variability in disease penetrance and phenotypic expression, posing challenges for risk prediction and clinical management. Additionally, multi-gene testing often identifies variants of uncertain significance or moderate-risk gene mutations not explicitly associated with HCRC [35,247]. Interpreting these results and determining management strategies is challenging, as clinical guidelines have not kept pace with the rapidly expanding list of susceptibility genes. Furthermore, high costs hinder patient access to genetic testing and treatment. In an expert survey on the implementation of genetic testing for CRC patients, 39% of respondents identified “lack of insurance coverage” as one of the main barriers to implementing population-wide genetic testing [248]. Precision medicine relies on genetic testing and molecular diagnostics, but many regions lack the necessary laboratories and specialized personnel. In low- and middle-income countries, limited access to biotechnology resources and insufficient genetic training among healthcare providers are identified as major barriers [248,249]. Genetic testing involves complex informed consent processes and raises ethical issues regarding how to handle unexpected genetic information and the potential for genetic discrimination [250,251]. These barriers often overlap, further complicating the implementation of precision medicine. In summary, we propose providing professional guidance to patients undergoing genetic counseling and genetic testing to help them understand complex genetic reports and various medical options [252]. Genetic testing and high-risk population interventions should be included in public health insurance and commercial insurance coverage, while strengthening professional training and international cooperation, and improving relevant laws and regulations.

Because hereditary cancers affect entire families and lifelong health, the role of communities and support networks in patient well-being is becoming increasingly prominent [253,254]. Some studies have established regular support groups for HCRC patients, where participants not only receive the latest medical knowledge but also share their psychological experiences during long-term screening and preventive surgeries [255]. Precision medicine is redefining the doctor–patient relationship, with doctors transitioning from mere decision-makers to information providers and consultants, while patients shift from passive recipients to active participants.

Despite the significant challenges posed by HCRC in accurately identifying high-risk populations and optimizing screening strategies, immunotherapy, personalized surgical strategies, and risk-oriented interventions have made significant progress in this field [155,256]. These advances have greatly improved treatment outcomes for HCRC patients. Meanwhile, AI, multi-omics, and liquid biopsy are playing an increasingly important role in early diagnosis and prognosis assessment [257,258]. However, issues such as insufficient screening adherence and the limited clinical application of precise predictive models remain prominent and urgently need to be addressed. Looking ahead, the deep integration of multi-omics and AI is expected to advance further the development of more precise and efficient treatment strategies. Researchers can use AI to collaboratively analyze massive datasets, such as genomes, transcriptomes, and proteomes, to overcome the challenges posed by HCRC’s high heterogeneity. Precision screening based on risk stratification can allocate screening benefits to high-risk populations, prioritizing high-risk individuals for enhanced screening or preventive interventions, while low-risk individuals can undergo non-invasive screening methods. A refined HCRC management system is anticipated in the future, which will significantly improve patient outcomes and advance the clinical translation of precision medicine.

## Figures and Tables

**Table 1:** Key Genes in HCRC MGPT.

Gene	Features	Syndrome	Mechanism	CRC Risk	References
*ATM*	Serine-threonine kinase	No typical syndrome	Germline mutations cause defects in DNA double-strand break repair and damage checkpoint function.	Low risk	Hall et al., 2021 [43] Xu et al., 2023 [44] Tuya et al., 2025 [45]
*CHEK2*	Serine-threonine kinase	No typical syndrome	Embryonic heterozygous mutations reduce the ability of cells to detect and repair double-strand breaks in DNA.	Low risk	Bychkovsky et al., 2022 [46] Erin et al., 2023 [47]
*BRCA2*	Tumor suppressor	Hereditary ovarian cancer syndrome	Germline mutations impair homologous recombination repair functions, leading to increased genomic instability.	Low risk	Momozawa et al., 2022 [48]
*NTHL1*	DNA glycosylase	*NTHL1*-associated polyposis syndrome	Embryonic double allelic nonsense mutations cause base excision repair defects, accumulating C:G to T:A mutations.	High risk	Belhadj et al., 2019 [49] Magrin et al., 2021 [50]
*POLE*	DNA polymerase ε	Polymerase proofreading-associated polyposis	Exonic enzyme domain germline mutations cause defects in DNA replication proofreading function.	Medium risk	Wang et al., 2023 [51]
*POLD1*	DNA polymerase δ	Polymerase proofreading-associated polyposis	Exonic enzyme domain germline mutations cause defects in DNA replication proofreading function.	Medium risk	Mur et al., 2020 [52]
*MLH1*	DNA mismatch repair enzyme	Lynch syndrome	Embryonic mutations cause MMR function defects.	High risk	Valle et al., 2019 [53]
*MSH2*	DNA mismatch repair enzyme	Lynch syndrome	Embryonic mutations cause MMR function defects.	High risk	Valle et al., 2016 [54] Valle et al., 2019 [53]
*MSH6*	DNA mismatch repair enzyme	Lynch syndrome	Embryonic mutations cause MMR function defects.	Medium risk	Valle et al., 2019 [53]
*PMS2*	DNA mismatch repair enzyme	Lynch syndrome	Embryonic mutations cause MMR function defects.	Medium risk	Hampel et al., 2006 [55] Ward et al., 2013 [56]
*EPCAM*	Cell adhesion molecules	Lynch syndrome	Heterozygous deletion of the 3′ end of the *EPCAM* gene can lead to hypermethylation of the adjacent *MSH2* promoter.	High risk	Hampel et al., 2006 [55] Ward et al., 2013 [56]
*APC*	Tumor suppressor	Familial adenomatous polyposis	Embryonic mutations cause *APC* inactivation, which prevents the suppression of β-catenin.	High risk	Li et al., 2016 [57]
*MUTYH*	Base excision repair enzyme	*MUTYH*-associated polyposis	Embryonic double-allele mutations cause defects in DNA oxidative damage repair, leading to the accumulation of G:C to T:A transitions.	High risk	Thet et al., 2024 [58]
*MSH3*	DNA mismatch repair protein	*MSH3*-associated adenomatous polyposis	Embryonic double allelic mutation causing *MSH3* deficiency.	Low risk	Taupin et al., 2015 [59] Yan et al., 2017 [60]
*AXIN2*	Wnt signaling regulator	*AXIN2*-related oligodontia	Nonsense mutations in the germline can cause Wnt signaling dysregulation.	Low risk	Lammi et al., 2004 [61] Bergendal et al., 2011 [62] Rebuzzi et al., 2023 [63]
*GREM1*	Secretory antagonistic factor	Hereditary mixed polyposis syndrome	A 40 kb tandem duplication upstream of the *GREM1* gene in the 15q13.3 region leads to increased *GREM1* expression.	Medium risk	Jaeger et al., 2012 [64]
*RNF43*	E3 ubiquitin ligase	Serrated polyposis syndrome	Embryonic lineage truncation mutation causes *RNF43* inactivation and Wnt signaling overactivation.	Low risk	Jaeger et al., 2012 [64]
*STK11*	Serine-threonine kinase	Peutz–Jeghers syndrome	Embryonic mutations cause *STK11* inactivation.	Medium risk	Rebuzzi et al., 2023 [63]
*SMAD4*	Signal transduction protein	Juvenile polyposis syndrome	Embryonic mutations cause TGF-β signaling pathway disruption.	Medium risk	Rashid et al., 2000 [65] Allen et al., 2003 [66]
*BMPR1A*	Bone morphogenetic protein receptor	Juvenile polyposis syndrome	Embryonic mutations cause BMP signaling damage.	Medium risk	Cao et al., 2006 [67] Lorans et al., 2018 [68]
*PTEN*	Phosphatase	Cowden syndrome	Embryonic mutations cause *PTEN* dysfunction.	Low risk	Syngal et al., 2015 [69] Rebuzzi et al., 2023 [63]
*TP53*	Transcription factor	Li-Fraumeni syndrome	Embryonic mutations cause *TP53* inactivation, resulting in the absence of p53-mediated cell cycle checkpoints and apoptosis mechanisms.	Low risk	Chung, 2018 [70]
*RPS20*	Ribosomal protein S20	Familial colorectal cancer	Embryonic truncation mutation causes loss of function of ribosomal protein S20.	Low risk	Nieminen et al., 2014 [71]

**Table 2:** HCRC molecular targeted therapy.

Target Gene	Mutation Type	Targeted Drugs	Therapeutic Effect	References
BRAF	V600E	Encorafenib + Cetuximab	ORR ≈ 19.5%, mPFS ≈ 4.3 months	Tabernero et al., 2021 [182]
KRAS	G12C	Adagrasib + Cetuximab	ORR ≈ 46%, mPFS ≈ 6.9 months	Yaeger et al., 2023 [183]
HER2	Gene overexpression	Tucatinib + Trastuzumab	ORR ≈ 38%, mDoR 12.4 months	J. Casak et al., 2023 [184]
NTRK	Fusion gene	Larotrectinib	ORR ≈ 79%, with some patients achieving CR	S. Hong et al., 2020 [186]

## Data Availability

No new data were created or analyzed in this study.

## Abbreviations

The following abbreviations are used in this manuscript:

HCRC	Hereditary colorectal cancer
MGPT	Multi-gene panel testing
ctDNA	circulating tumor DNA
dMMR	Mismatch repair deficiency
AUC	Area under the curve
CRC	Colorectal cancer
FAP	Familial adenomatous polyposis
IHC	Immunohistochemistry
MMR	Mismatch repair
MSI	Microsatellite instability
NGS	Next-generation sequencing
ORR	Objective remission rates
PCR	Polymerase chain reaction
PVs	Pathogenic variants
WSI	Whole slide imaging
AI	Artificial intelligence
LS	Lynch syndrome
OS	Overall survival
